# Is Protecting Older Adults from COVID‐19 Ageism? A Comparative Cross‐cultural Constructive Grounded Theory from the United Kingdom and Colombia

**DOI:** 10.1111/josi.12538

**Published:** 2022-08-17

**Authors:** Elfriede Derrer‐Merk, Maria‐Fernanda Reyes‐Rodriguez, Ana‐Maria Salazar, Marisol Guevara, Gabriela Rodríguez, Ana‐María Fonseca, Nicolas Camacho, Scott Ferson, Adam Mannis, Richard P Bentall, Kate M Bennett

**Affiliations:** ^1^ University of Liverpool Liverpool UK; ^2^ Universidad El Bosque Bogotá Colombia; ^3^ University of Sheffield Sheffield UK

## Abstract

The COVID‐19 pandemic impacted people's lives all over the world, requiring health and safety measures intended to stop the virus from spreading. This study explores whether an unintended consequence of these measures is a new form of ageism. We explore, using qualitative methods, the experiences of older adults living through the pandemic in the United Kingdom and Colombia. Although there were some small differences between countries, for the most part, the experiences were similar. We found that older adults reported that they were seen as a homogenous group and experienced both benevolent and hostile ageism and a loss of autonomy as a consequence of COVID‐19 protection measures. Participants from both countries expressed anger and frustration, and increased anxiety, and felt that their individuality was ignored. We recommend that policy‐makers, the media, and wider society consider the impact of such health and safety measures on older adults in preparing for future pandemics and health challenges.

## INTRODUCTION

Late in 2019, the world^1^ was shocked by the arrival of a new severe acute respiratory coronavirus (SARS‐CoV‐2, known as COVID‐19 disease) detected in Wuhan, China. The lives of the majority of the world's population have been disrupted (Singh et. al., 2020; Department of Health and Social Care [DSHC], 2020a). Management of the pandemic has depended on countries’ economic capacities and public health systems. The pandemic affected different regions of the world unequally, and governments have taken different measures to control it, depending on their economic, social, and cultural characteristics and capacities (Ahmed et al., 2020; Garcia et al., 2020). For example, Colombia had one of the longest lockdowns of 2020 (Austria, 2020). These decisions were made as a measure to control the pandemic whilst the health care system was strengthened; in 2020, Colombia doubled the ICU bed capacity (MinSalud, 2020). Many Latin‐American countries (Argentina, Brazil, Colombia, Chile, Ecuador, Mexico, and Uruguay) established specific lockdowns and restrictions for individuals over 60 or 70 years and stated that those measurements are to protect older adult's lives (Acosta et al., 2021).

This paper focuses on two countries with different characteristics: The United Kingdom (UK) and Colombia. First, we outline the situations in each country and then discuss the literature on ageism in relationship to the pandemic.

### UK

A nationwide lockdown was implemented in England on the 23rd of March 2020 and lasted 12 weeks (Cabinet Office, 2020). Health‐related decisions were devolved to the four constituent nations, Wales, Scotland, England, and Northern Ireland, who imposed similar lockdowns. People were not permitted to leave their homes except for once‐daily exercise or essential trips, like grocery shopping or health‐related activity. Those categorized as ‘at greater risk’ of developing serious complications if they got the virus were contacted by their general practitioner to be advised not to leave the home (called shielding) (Department of Health and Social Care [DHSC], 2020a). All people aged 70 and over (BBC, 2020a) were strongly advised to stay at home for 12 weeks between 20th of March 2020 until June 2020 and not meet anybody from outside their immediate household unless essential. In July 2020 although new policies were implemented for people who were advised to shield, they were no longer age‐related.

Across the world the pandemic has particularly impacted the lives of older adults as COVID‐19 causes them more severe morbidity and greater mortality than other age groups (Crimmins, 2020; Dowd et al., 2020). Thus, in the UK it was hypothesized that older adults might experience a marked impact on their psychological and social wellbeing in addition to the impact on their physical health (Department of Health and Social Care [DHSC], 2020b). Shielding policies were extended to care homes, where many residents were older, making it impossible for relatives to visit their now isolated family members. Statements by Prime Minister Boris Johnson such as “many more people will lose loved ones” seemed to accept that people would die from the virus and that deaths were unavoidable (Reuters, 12th March 2020). This approach presumed a pandemic response based on herd immunity (BBC ITV, 2000), which was quickly reversed by the government.

### Colombia

Latin America is a region with high social inequality among population groups, although this varies significantly from country to country. Despite this diversity, the region shares a context of institutional weakness and low social security coverage for older adults (Centro Latinoamericano y Caribeño de Demografía [CELADE], 2009; Redondo, 2009). Unfortunately, because of political instability in Latin America, the pandemic has disproportionately affected the region (Garcia et al., 2020).

On March 12, the government declared a public health emergency and the first lockdowns and quarantines began. The government subsequently implemented diverse sanitary, health emergency measures, and social and economic actions (Ministerio de Salud y Protección Social [MSPS], 2020). On March 20, 2020, the Colombian government introduced *The Mandatory Health Measure of Preventive Isolation*, for people over 70 years of age, which was imposed originally until May 30, 2020 but then extended through August 2020 as a way of protecting older adults against the virus. Under these measures, centers that offered activities for older individuals, including adult day‐care centers, were closed (MSPS, 2020).

In June 2020, it was determined that “adults over 70 years of age could perform physical activities and exercise three times per week, half an hour a day” (MSPS, 2020, p. 4). Colombia has since gradually implemented a protocol allowing free movement of older adults in open spaces complying with certain criteria to protect individuals who suffer from chronic diseases (MSPS, 2020). These measures were controversial among the older adult population. A group of older adults alleged that mandatory preventive isolation violated their constitutional rights to equality, freedom of movement, and free development of the personality. As a consequence, it was decided to use persuasion through self‐care recommendations in recognition that older adults could understand and make decisions on their own (Ámbito Jurídico Legis, 2020). Since then, the government has implemented different quarantines and lockdowns, restrictions on gatherings of the general population without any exception by age or any other category.

### Extraordinary situations call for extraordinary research

COVID‐19 challenged not only individuals in day‐to‐day lifestyle and activity, it also challenged researchers worldwide. This led to a dash to gather data and naturally, because of the different course of the pandemic in different countries, the timing of research projects also differed. Although this makes cross‐cultural comparisons more challenging, it is important that these studies take place. We argue, therefore, that “different research interests call for slightly different methodological research designs” (Halkier, 2017, p. 1). Our existing Anglo‐Colombian collaboration provided a unique opportunity to investigate the lived experiences of older adults during the COVID‐19 pandemic as a researcher (Charmaz, 2014; Karasz & Singelis, 2009). In using constructivist grounded theory, our intention is to develop theory, rather than to test existing theories. As such our findings are grounded in the language of our participants, rather than in academic terminology (Charmaz & Thornberg, 2021).

**FIGURE 1:**
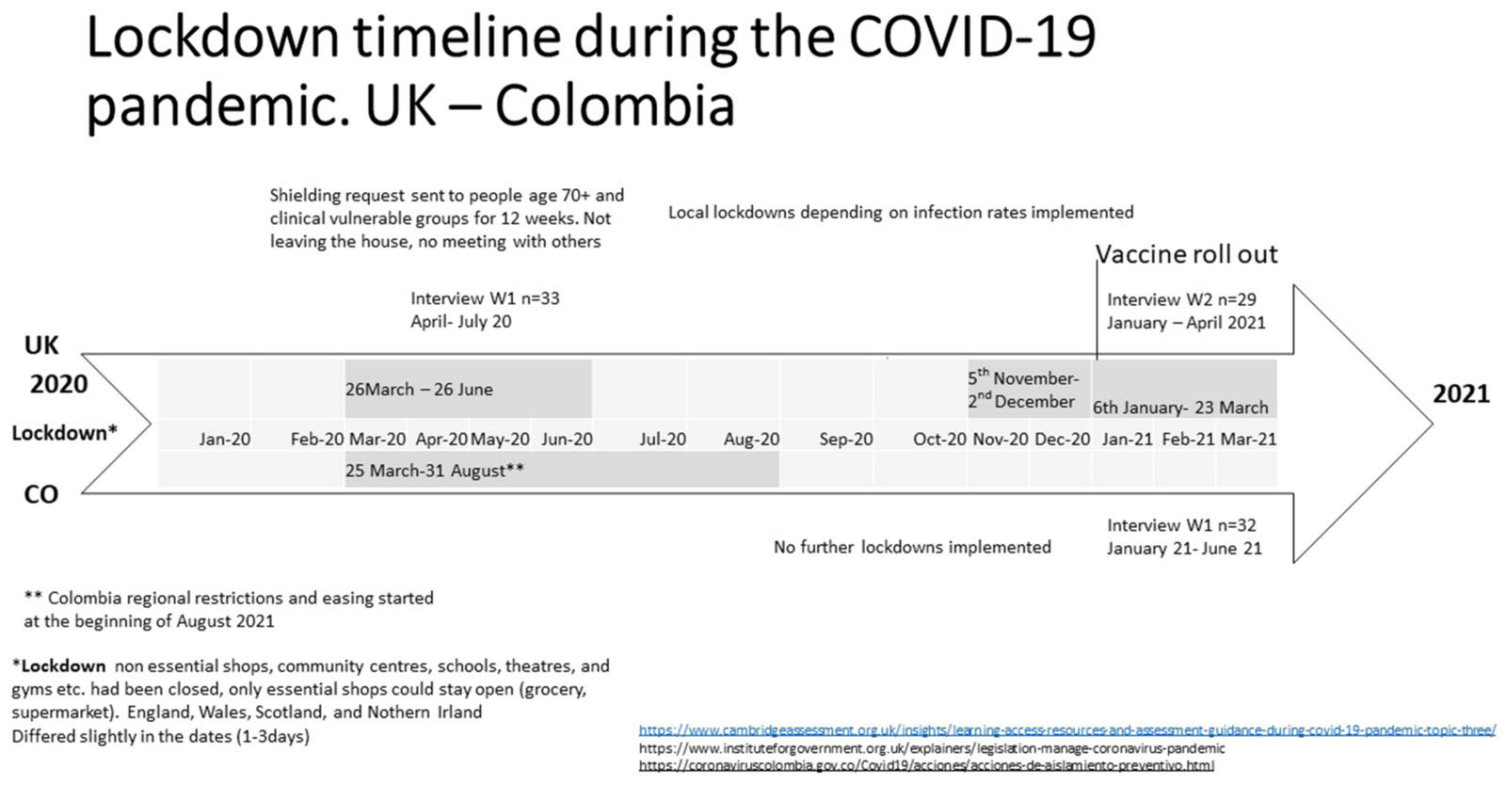
Lockdown timeline comparison UK and Colombia (January 2020 to March 2021) [Colour figure can be viewed at wileyonlinelibrary.com]

Conducting cross‐cultural studies during a pandemic requires a creative and flexible approach (Karasz & Singelis, 2009). Recruitment, financial and research resources, interview time‐points, analyses, teamwork, and language constraints all require a flexible approach. For example, as a consequence of these challenges, we interviewed participants at different time‐points but about lockdowns, which occurred at broadly the same time (Figure 1).

The figure demonstrates that the initial lockdown in both countries took place with slightly different length at the same time. This is the common ground, which enabled us to compare both countries in this study. The research question and question in the interviews focused on the experience of age‐related discrimination. The age discrimination from the participants experience in turn focused on the new COVID‐19 restrictions (shielding, stay at home, not leave the house), which had been implemented in both countries in March 2020.

The epistemologies, ideas, and individual ontology determine the way in which a study can be judged on the quality (Charmaz & Thornberg, 2021). Long et al. (2020) states that there is not “one size fits all” strategy for critical appraisal. Corbin and Strauss (2015) argue, “little consensus about what constitutes an appropriate set of evaluation criteria for qualitative research” (Corbin & Strauss, 2015, p. 341). However, we focused in this study on the criteria given by constructivist grounded theory (Charmaz & Thornberg, 2021). These are: credibility, originality, resonance, and usefulness.

### Ageism and the pandemic

The COVID‐19 pandemic forced countries across the world to change national policies to control the spread of the virus in order to protect older adults and vulnerable people. The intention to protect towards older adults was promoted in both countries (Hodgson & Peytrignet, 2021; MinSalud, 2020)

Many of these policies, such as shielding advice or new laws to stay at home, may constitute age discrimination. Further, informal discrimination may also have been propagated worldwide via media and social media. These structural discriminations may establish a new form of ageism. Country‐specific advice in the UK and law in Colombia confined older adults age 70+ during the lockdown(s) and beyond to their home. This may be a new structural ageism enabled through the health and safety measures.

A historical analysis from the United States of America (USA) demonstrates how the change of stereotypes over five decades impacts society's attitudes and in consequence policy making and media reports (Binstock, 2010). He identified that the stereotype of vulnerability of older adults led to policies to support older adults and described it as “compassionate ageism.” This attitude changed over time when government's financial resources and priorities changed and saw older adults as “greedy geezers” and later in time to the concern of societies overtaking “senior power” in elections and policy‐making, namely health care and entitlement programs (see also in this issue Kanik et al., 2022). Arrubla (2014) analyzed the social policies aimed at the elderly from 1970 to 2009 in Colombia. She suggests that during these period of time social policies under the demographic discourse have generated new forms of ageism and legitimized through the discourse of dependency and fragility.

Recent North American research found that positive responses to older adults during the pandemic increased their sense of value and improved their mental health and well‐being, whereas negative responses had the opposite effect; they noted that some positive responses (e.g., protection) might have negative consequences such as increased loneliness (Monahan et al., 2020). Benevolent ageism has recently been recognized as a form of patronizing and over‐accommodating attitude towards older adults based on an age stereotype of impaired competence. Sublett and Bisconti (2020) found that this kind of ageism adversely impacted on older adults’ self‐compassion, which in turn influenced their appraisals of their cognitive abilities. People who had fewer experiences of benevolent ageism were found to have higher rates of self‐compassion and this resulted in better evaluations of their memory. Similarly, Visintin (2021) in Italy found that, during the pandemic, containment behaviors were positively associated with the quality of contact with older adults and with benevolent ageism. In the USA, research found that “Higher hostile ageism predicted lower pandemic‐related behavior modification. Those high in benevolent ageism reported lower behavior change, but also reported higher pandemic‐related fear” (Vale et al., 2020, p. 1). Swift and Chasteen (2021) found that the discourse about the pandemic strengthened the view of British older adults as a homogenous group and consequently stigmatized them. In Canada, stigmatization leads to new ways of coping for older adults, for example, feeling younger than the compared age group and highlights the phenomenon of subjective age bias (Chasteen & Cary, 2015). In a systematic review, Chang et al. (2020) found that structural and individual levels of ageism independently have an adverse effect on health.

Bravo‐Segal and Villar (2020) found that 74% of the news headlines in Spain had stereotyping and ageist information when referring to COVID‐19 and older adults. In Latin‐American, HelpAge International (2020) found that portraying COVID‐19 as a disease of older population was generating ageism and legitimizing stereotypes.

Ehni and Wahl (2020) in Germany, developed a series of propositions about how to fight ageism during the pandemic. These were: it is important to recognize the heterogeneity of older adults; age limits of medical support are unethical; mass deficit views of old age are dangerous; intergenerational solidarity must be supported; paternalistic assumptions must be resisted; the use of new technology among older adults should be fostered; and finally, knowledge of gerontology should inform policymaking (see also in this issue Lytle & Levy, 2022; Drury et al., 2022; Jarrott et al., 2022; Ng et al., 2022; Sutter et al., 2022). In addition, attention has been drawn to digital exclusion (Sourbati & Behrendt, 2020; Rosales & Fernández‐Ardèvol, 2020) and enforced isolation (Fletcher, 2021) as forms of ageism. More recent studies suggest that combatting ageism is possible for example, Lytle and Levy (2022) found in an experimental design, that ageism reduction interventions were successful. (see also Ayalon & Okun, 2022; Montepare & Brown, 2022).

### Purpose of the present study

The need for and challenges of cross‐cultural studies has been discussed in general, by Kline et al. (2018) and Broesch et al. (2020). This study enriches the knowledge about experiences during the pandemic not only for Western, educated, industrialized, rich, and democratic (WEIRD, Henrich et al., 2010) countries, but also for developing countries like Colombia. The UK and Colombia are economically and socially distinct countries in different continents; the UK is considerably richer with a per capita GDP of $46,659 compared to Colombia's of $14,722 (https://www.cia.gov/the‐world‐factbook/field/real‐gdp‐per‐capita/country‐comparison, Central intelligence agency [CIA], n.d.). Life expectancy in the UK is 81.3 years compared to 76.91 in Colombia. Unemployment is considerably lower in the UK (3.17% as opposed to 10.5%) and education expenditure is higher (5.4% of GDP compared to 4.5%). Many older Colombians live below the UN poverty line (Bennett et al., 2016) (Table 1).

**TABLE 1:** Sociodemographic data UK and Colombia (January 2022)

**Data**	**UK**	**Colombia**
Population	66 million	49.1 million
Live expectancy	81.3 years	76.91 years
People age 65 and over	18%	31%
GDP per capita	$ 43K	$ 6.7K
Unemployment	3.17%	10.5%
Education expenditure	5.4%	4.5%
Living under the UN poverty line	Total 12,4%, age 66+ 15.5%,	Total 35.7%, age 66+ 28%
Internet access (2018)	94.9%	62.3%
Health care	NHS	2017 94.41% (45.5 m) covered with insurance
ICU beds per 1000 inhabitants	2.4	1.48
COVID‐19 death per one million population March 14, 2022	2422.48	2765.01

https://databank.worldbank.org/data/download/poverty/987B9C90‐CB9F‐4D93‐AE8C‐750588BF00QA/AM2020/Global_POVEQ_COL.pdf, https://www.statista.com/statistics/1104709/coronavirus‐deaths‐worldwide‐per‐million‐inhabitants/, https://equityhealthj.biome2central.com/articles/10.1186/s12939‐020‐01241‐0, https://www.mylifeelsewhere.com/compare/colombia/united‐kingdom, https://data.oecd.org/healtheqt/hospital‐beds.htm, https://covid19.who.int/region/euro/country/gb, https://covid19.who.int/region/amro/country/co

To our knowledge, there has been no qualitative, cross‐cultural study about the experiences of older adults of the COVID‐19 pandemic comparing the UK and Colombia.

This paper examines the impact of COVID‐19 on ageism in both the UK and Colombia, focusing on both similarities and differences. In this paper, we are focusing on the question: Did policies aimed at protecting older adults from COVID‐19 establish new forms of ageism?

## DESIGN AND METHODOLOGY

An exploratory qualitative research project was carried out using the constructivist Grounded Theory approach of Charmaz (2008, 2014). We developed a unique cross‐cultural comparison between the UK and Colombia, by collecting data in both countries with the same interview method, with minor differences based on language adaptations and on the different dates that the data were collected.

The CPR19, formed in March 2020, aimed to investigate the psychological, social, and economic impacts of the COVID‐19 pandemic (https://www.sheffield.ac.uk/psychology‐consortium‐covid19). This longitudinal, internet panel survey assessed: (1) COVID‐related knowledge, attitudes, and behaviors; (2) the occurrence of common mental health disorders; as well as the role of (3) psychological factors; and (4) social and political attitudes in influencing the public's response to the pandemic. Quota sampling was used to recruit a nationally representative (age, sex, and household income) sample of adults (*N* = 2025) (McBride et al., 2021). The consortium was not confined to the UK but included initial collaborations from Northern Ireland, Italy, and Spain. Building on an existing collaboration between (last author) (UK) and (second author) (Colombia), it was agreed that qualitative data collection be extended to Colombia using the same methodology (details later). The first and second authors had been trained both in the analytical process of constructivist grounded theory development by the senior author, who has more than 20 years of qualitative experience, ensuring consistency and quality of analysis. Once the schedule and sampling strategy was agreed, the two studies proceeded independently, taking account of the different timelines of the pandemic.

### Researchers

Derrer‐Merk, Reyes‐Rodriguez, and Bennett are qualitative researchers with expertise in the psychological experiences of ageing and resilience. Salazar is an expert in clinical psychogerontology and supported the writing. Ferson and Mannis supported the initial design and writing. Bentall is an expert in the psychology of mental health and the Principal Investigator of the UK arm of the C19PRC and contributed to the design and writing. We supported capacity building for the Colombian psychology undergraduate students Guevara, Rodríguez, Fonseca, and Camacho. They were involved in the data collection and analysis.

### Participants

The participants from the UK were adults aged 65 and over. Altogether 33 participants took part in the interview: six men living alone, nine men not living alone, 12 women living alone, six women not living alone. (age range 65–83, mean = 71, SD = 5) (Table 2).

**TABLE 2:** Demographic data from Colombia and the United Kingdom

	UK			Colombia		
	ID	Age	Marital status	ID	Age	Marital status
Male living alone	M1	67	Widowed	SH01	67	Never married
	M3	74	Never married	SH02	63	Divorced
	M7	83	Divorced	SH03	79	Widowed
	M8	73	Divorced	SH04	68	Divorced
	M10	75	Divorced	SH05	64	Never married
	M14	77	Widowed	SH06	87	Widowed
				SH07	66	Never married
				SH08	62	Never married
Male not living alone	M2	73	Married	AH01	64	Married
	M4	71	Married	AH02	64	Married
	M5	75	Married	AH03	62	Never married
	M6	71	Married	AH04	65	Married
	M9	64	Married	AH05	66	Married
	M11	70	Married	AH06	64	Married
	M12	66	Married	AH07	66	Married
	M13	77	Civil partnership	AH08	66	Married
	M15	72	Married			
Female living alone	F2	78	Never married	SM01	67	Divorced
	F4	75	Widowed	SM02	66	Never married
	F6	67	Widowed	SM03	83	Widowed
	F8	66	Separated	SM04	84	Widowed
	F9	82	Widowed	SM05	66	Widowed
	F10	65	Never married	SM06	95	Never married
	F11	73	Divorced	SM07	84	Widowed
	F12	68	Divorced	SM08	72	Never married
	F14	76	Separated but still married			
	F15	73	Divorced			
	F16	73	Divorced			
	F17	70	Widowed			
Female not living alone	F1	65	Married	AM01	68	Married
	F3	71	Married	AM02	62	Married
	F5	65	Divorced	AM03	67	Never married
	F7	65	Married	AM04	71	Married
	F13	68	Married	AM05	68	Married
	F18	66	Married	AM06	61	Married
				AM07	64	Married
				AM08	70	Married

To reach a similar sample of participants the researchers in Colombia recruited 32 participants aged 60 years and older (range 63–95; mean = 69, SD = 9). The sample of 32 people consisted of eight men who live alone, eight men who do not live alone, eight women who live alone, and eight women who do not live alone (Table 2).

### Recruiting procedures

Participants in the UK were recruited from the CPR19 study. Participants from the main study were asked if they would be willing to be approached to participate in add‐on studies and those who consented were grouped in three subsamples and contacted by email to ask if they would be willing to be interviewed: older adults (≥65 years old); adults (18–64); and pregnant women and parents with children under the age of 1 year old. This paper focuses on the sample of older adults. In the larger study, there were 287 participants aged 65 and over and we contacted 86 of those, and 33 participants agreed to participate (38.4% participation rate). The interview team approached potential participants in batches of 5–10 and ceased recruitment once theme saturation had been reached. The first author contacted 26/33 participants, the seven remaining participants were interviewed by Liverpool colleagues. Interviews were conducted between April and July 2020. Participants from the UK were compensated with a £10.00 voucher.

The research in Colombia was entirely qualitative, and the participants were recruited using a snowballing sampling approach. Participants were contacted through social networks, emails, and “word of mouth.” Interviews were conducted between January and April 2021. No compensation was provided.

### Ethical approval for the UK and Colombia

The UK's ethical approval for the national representative study was granted by the University of Sheffield University (ref: 033759). The qualitative substudy was approved by the University of Liverpool (ref: 7632‐7628). The Universidad El Bosque, Bogota‐Colombia received ethical approval for the qualitative study (ref: 002‐2021).

### Data collection

The data collection was undertaken using a semistructured interview schedule, which was the same in both countries, via in‐depth interviews. Interviews were undertaken remotely via telephone, or by remote conferencing platforms (audio only), and lasted between 30 and 90 min. We asked the participants how they would like to be interviewed and they chose their preferred interview method. We did not ask about the technical accessibility nor the ability to use it. Our sample, unfortunately, did not include digitally excluded participants. The interviews were audio‐recorded and externally transcribed verbatim in both countries. Before beginning the interview, respondents were sent an information sheet and a consent form to read and sign. Confidentiality and anonymity were assured. The interviews were not tightly structured, rather the aim was to learn what was important to the participants. The approach takes *“participants' views and voices as integral to the analysis––and it's presentation”* (sic) (Charmaz, 2008, p. 402). Two broad questions were asked, “how did you feel?” and “what did you do?” The interviews led participants chronologically through their experiences of the COVID‐19 pandemic. They were asked about their lives prior to the pandemic, and then about their lives during the pandemic, and finally about their thoughts about their future after the pandemic. They were also asked their views about their communities, local and central government, and social and traditional media. We also asked about their experiences as older adults, and whether, for example, they had experienced discrimination either before or during the pandemic, and how this might change post‐pandemic. Finally, they were asked what advice they would give to others in the same situation.

All the interviews were conducted in the participants’ native languages–Spanish for Colombia and English for the UK. The Spanish quotes used in this paper were translated into English and can be seen in the original language (Table 3). Some of the Spanish quotes appear awkward to the English ear. However, as we wanted to preserve the sense and feeling of the interviews, we have chosen to present literal rather than grammatically corrected quotes for both the English and the translated Spanish. The gold standard of back translation had been undertaken by colleagues from the Universidad el Bosque.

**TABLE 3:** Translated quotes from Colombia

English	Spanish
CO SH04 "Right now the government has had a very important interest in the elderly (…) to avoid high mortality, and I think that if there has been more interest than before and it has made others think more about the older adults.”	“Ahorita el gobierno ha tenido un interés importantísimo en los mayores (…) para evitar la alta mortalidad y creo que si se ha visto más interés porque antes no y nos ha puesto a pensar más en los adultos mayores”
CO SH05 stated "It was a lot of confinement … it gave me a lot of monotony, boredom, and made me sick (…) That isolation factor seemed cruel and severe to me."	“Mucho encierro…me dio mucha monotonía, aburrimiento y me enferme…Ese factor de aislamiento me parecía cruel y severo”
CO SM08 “it was an act disguised as kindness, but that was discriminatory, it was when the elderly could not go out and could not do anything (…) no, the older citizens are just a figure for many (government).”	"Fue un acto disfrazado de bondad, pero que era discriminatorio fue cuando los adultos mayores no podían salir y que no podía hacer nada (…) no, el adulto mayor es una cifra más para muchos"
CO AM04 “When they began to say that adults over 70 could not go out, then it felt discriminated against, like you have to take care of them more … I know that taking care of you is a delight, but when they tell you, I felt little discrimination.”	"Cuando empezaron a decir que los adultos mayores a 70 no podíamos salir, entonces se siente como discriminado, como que hay que cuidarlos más… yo se que lo cuiden a uno es una delicia, pero cuando le dicen, si siento un poco de discriminación"
CO SH02 "the thing is that the pandemic is hitting mainly older people, then now define what the term ‘older people’ means. So now, older people refer to 60 and up, then they change the term for senior to older people or elderly, those are the ones at higher risk, crap! I think it was mismanaged.”	"no es que la pandemia le esta pegando más a los viejitos, entonces ahora defina viejitos, entonces ya viejito es el de mayor de 60, entonces ahora ya te cambian el tema de adulto mayor por el de viejito ya, y el de más riesgo, entonces juepucha eso fue algo que creo yo que no se supo manejar"
CO LAH02 “The president said to the ‘grandparents’, that we have to lock down and I don't know why it has to be mandatory, that is a lack of respect, a sovereign lack of respect for that asshole (…).”	"el señor presidente dijo que los 'abuelitos' nos tenemos que encerrar y no se qué, eso es una falta de respeto, una soberana falta de respeto de ese pendejo"
CO AM06 “The truth is like what I tell you, I don't feel like an older adult yet, but let's say, when everything started with the pandemic, all those over 60 years of age and older, nobody can go out then, as there I said oh no I am older adult? as I am already old? but no!”	"la verdad es como lo que te digo como que aún no me siento adulta mayor, pero digamos cuando empezó todo lo de la pandemia todos los mayores de 60 años en adelante nadie pueden salir entonces como que ahí dije brutas como que yo ya estoy viejita pero no"
CO SH02 "oh no, that you have to take care of them (older adults) (…), at this age one has more responsibility and one knows to care better than any 20 year old kid (…) they annoyed me (…).”	"ay no que hay que cuidarlo que no se que, no nada, uno a esta edad (tiene) es más la responsabilidad y uno se sabe cuidar más que cualquier pelado de 20 años (…) pero tampoco es para que lo jodan a uno (…) noo tampoco, ya"
CO SHO7"They (the government) should give us a voice, they have to respect their rights and not steal the money that is for the older adults.” SH07	"que le den voz, que les respeten los derechos y que no se roben la plata que es para el adulto mayor"
CO AH03 “the pandemic made us visible … The president by a mandatory lockdown also made an acknowledgement that we existed, and later we had to reveal (legal action) ourselves so that they let us release from the lockdown, because it was too excessive (lockdown).”	"La pandemia nos hizo visibles…el señor presidente al hacernos guardar por obligación también hizo un reconocimiento de que existíamos, ya después nos tocó revelarnos para que nos dejaron salir si porque ya fue en exceso"

### Analysis

The authors’ ontological and epistemological standing is grounded in the belief that there are multiple truths and realities about phenomena and that society and humans construct their own reality, which needs to be acknowledged and valued. This is congruent with Charmaz (2014, p. 17): “*I assume that neither data nor theories are discovered either as given in the data or the analysis. Rather, we are part of the world we study, the data we collect and the analyses we produce*.” This qualitative, constructivist grounded theory approach is appropriate for exploring the experience of older adults during the COVID‐19 pandemic since it accounts for both the participant's and the researcher's experiences and perspectives (Bennett & Vidal‐Hall, 2000; Charmaz, 2014, 2020). The analysis is inductive and iterative, using constant comparison. The analysis was undertaken manually in MS Word and Excel. Interviews were coded and initially analyzed by researchers from the country of origin (team University of Liverpool, UK and team Universidad El Bosque, Colombia; and exchanged after the initial coding). Both teams discussed the findings in each step of the analytical process. First, interviews were read line‐by‐line to give a holistic impression, followed by line‐by‐line coding. This initial coding was summarized in a codebook, which included initial codes, the meaning of the codes, and quotes related to each participant. This process was reflective: as new topics emerged, they were looked for in earlier parts of the interview. Second, a more focused approach was employed to generate categories that emerged as particularly significant in the data. Once categories were extracted for each of the interviews, the transcripts were compared to identify broader themes and commonalities or diversities. Each coder wrote a memo helping to construct theoretical categories, and ensuring reflexivity and analytical thinking (Charmaz, 2014). Third, once initial coding and analysis had taken place, the first and second authors met regularly to discuss the analysis; the draft of the ageism abstract was available on google docs, where all authors were discussing findings and contributed to the final paper. The participants in the UK were interviewed a second time in January–March 2021 but this data is not included in the current study. Three central themes emerged from the data: benevolent versus hostile ageism; society's view of ageing as homogenous; and lost autonomy. In the presentation of the quotes, the country of origin is represented by CO (Colombia) and UK.

## FINDINGS

It is remarkable that the cross‐cultural analysis undertaken independently, and given the differences both in culture and in the timeline of the interviews, found similar results. Overall, the pandemic has exacerbated ageism, and in turn, that ageism has impacted the lives of older adults. We discuss the three main themes in turn: benevolent versus hostile ageism; ageing viewed as homogenous; and lost autonomy. As noted above, we provide literal (rather than grammatically correct) translations of the Spanish to English to reflect the context of the quotes.

### Benevolent versus hostile ageism

The first theme identifies two different views of ageism experienced by participants. Broadly participants either saw the ageism they experienced as benevolent, or as hostile (not mutually exclusive). Some participants perceived a benevolent ageism in the way that the pandemic was addressed and presented. They accepted and understood the public health measures as a positive recognition of the value of older adults. Conversely, other participants experienced these measures as hostile. They felt discriminated against because of their age. For example, participants reported that they were probably healthier than many younger people. This led older adults to perceive the measures as discriminatory, promoting exclusion and ageism.

*Benevolent ageism*. Some older participants in Colombia accepted their exclusion from society and participation in social life and viewed it as an act of love and protection. Some initially felt that these government measures were a symbolic recognition of older adult's existence, which they had never felt before.

Right now the government has had a very important interest in the elderly (…) to avoid high mortality, and I think that if there has been more interest than before and it has made others think more about the older adults (CO SH04).

This view was less common amongst UK older adults. Only a few participants felt privileged to be considered important by the UK government:

If at all, I think the Government is looking after older people. If there's any discrimination, it's positive discrimination rather than anything else (UK F17).

*Hostile ageism*. More common, especially as time progressed, was the view that both governments’ policies were ageist in a way that was detrimental. For example, in Colombia, when the duration of quarantine was extended for older adults, many started to feel that their rights to choose how they lived were being curtailed. They further believed that the isolation measures were too severe:

It was a lot of confinement … it gave me a lot of monotony, boredom, and made me sick (…) That isolation factor seemed cruel and severe to me (CO SH05).

Older adults developed critical thinking about their own risk and decision‐making about self‐care behaviors, despite understanding the rationale for governments’ policies. UK M12 assumed that the government had a hidden agenda. He explained how people with disabilities were considered to need special care in special homes, but the truth for him was that people with special needs need to be locked up as it is uncomfortable for the public to see people with disabilities. He questioned the motivation behind protecting older adults.

It is said to be for their protection, but then there's often things that society does for somebody that is claimed to be for somebody else's protection (UK M12).

One participant from Colombia discussed the discriminatory policy of the government:

It was an act disguised as kindness, but that was discriminatory, it was when the elderly could not go out and could not do anything (…) no, the older citizens are a figure for many (government) (CO SM08).

Additionally, the traditional media outlets and social media platforms were important sources of information and important for social connectivity, and this increased during the pandemic. Knowing that the virus might be more harmful to them, the participants were eager to learn how to protect themselves. However, the ongoing bad news in the press left them feeling anxious and increasingly depressed. Thus, the media were perceived to be promoting hostility as they reported bad news about the ageing population. These were signs of exclusion and countered the existing policies about ageism (Ehni & Wahl, 2020). As a result, many did not watch the news anymore as UK M8 discussed:

At first, watching the news every day is depressing and getting more and more depressing by the day, so I've had to stop watching it for my own peace of mind.

The necessity to use new technology also highlighted the digital divide experienced by some older adults. If older adults were not willing, or unable, to change their communication behavior, they were disadvantaged. UK M8 was sticking with the telephone or face‐to‐face communication and mentioned refusing computer conversations:

But it's not the same as face‐to‐face contact. With the best will in the world… I have no cameras on my computer, I won't do computer conversations, but face‐to‐face.

The shielding advice created practical difficulties, with shopping, communication, and health support. Shopping in person was no longer advised for people who were shielding in the UK, but an organized replacement from the UK government or community was problematic. Although the UK supermarkets offered new online deliveries and protected shopping hours (priority slots for older adults), this was not always available or desirable. Other participants needed to rely on neighbors or family (Derrer‐Merk et al., 2022). UK F18 discusses how stressful it had been at the beginning of the lockdown in March 2020 to organize essential shopping:

At first it was very, very stressful, I signed up to every supermarket known to man and tried to get deliveries and couldn't.

This problem was unique to the UK because in Colombia delivery services (via phone calls) were already part of the shopping culture pre‐pandemic.

In the UK as one participant had his health care package withdrawn with immediate effect in March 2020. Evidence from a physician that the care was still essential was required to resume his care support (UK M8). He said the experience was “horrific” and he had been left isolated:

As far as the care agency was concerned, I didn't meet the criteria, so I had my care withdrawn. (…) So, it's been a bit of a nightmare, to be honest.

Participants in Colombia spoke frankly about how they felt discriminated against by the policy to stay indoors:

When they began to say that adults over 70 could not go out, then it felt discriminated against, like you have to take care of them more … I know that taking care of you is a delight, but when they tell you, I felt little discrimination (CO AM04).

### Society's view on ageing as homogenous

In both countries, policies and recommendations were generalized to the older adults’ population, based on the assumption that all older adults age in the same way, without recognizing that ageing is heterogeneous. These generalized assumptions shaped political decisions and behavior. Defiant arguments against the homogeneous view of older adults were found in both samples. For example, from UK M12:

Clearly it can't be as black and white as that because there are some very fit 80‐year olds and there are some very unfit 50‐year olds so it shouldn't be anything to do with your age.

One participant, UK M4, was opposed to the need to be cocooned:

In terms of a lockdown continuing for anybody and I really would kick out against that because I don't think I fit the bill that needs to be cocooned yet. I possibly do medically, I don't know, but I don't mentally feel that I want to be cocooned.

The grouping of older adults as a unified whole in Colombia led to naming all older adults as “older people” or grandparents. One participant, CO SH02, discusses the meaning of naming and demonstrates her anger at how older adults are named:

The thing is that the pandemic is hitting mainly older people, then now define what the term ‘older people’ means. So now older people refer to 60 and up, then they change the term for senior to older people or elderly, those are the ones at higher risk, crap! I think it was mismanaged.

Additionally, being called “grandparent” offended Colombian participants:

The president said to the ‘grandparents’, that we have to lock down and I don't know why it has to be mandatory, that is a lack of respect, a sovereign lack of respect for that asshole (…) (COL AH02).

This homogeneous perspective on older adults led to a new experience of forced identity where participants felt discriminated against. Being even called an older “person”^2^ resulted in many cases in discussions about self‐perception. UK F14 shared her anger and disagreement about the age‐related stereotype and discrimination:

Well, first of all, a lot of us who are over 70 thought well why us because most of us are a lot fitter and healthier than a lot of people who are under 70 who are allowed to go out, so that seems to be arbitrary to start with.

Participants in both countries discussed how naming older “people”^2^ challenged the self‐perception of ageing. UK F16 draws the direct consequence of being named as old and feeling it:

Well, reduced mobility because of arthritis, that made me feel old and also I know this is before the lockdown but as soon as the government told us that we were elderly, I felt it.

Another participant in Colombia dismissed the idea of being named old. This applies to the participants from the UK as well as from Colombia. CO AM06 remarked:

The truth is like what I tell you, I don't feel like an older adult yet, but let's say, when everything started with the pandemic, all those over 60 years of age and older, nobody can go out then, as there I said oh no I am an older adult? as I am already old? but no!

### Lost autonomy

Participants who had to stay at home sacrificed their freedom for the benefit of society. This policy and its implications were widely criticized among the participants. They believed that their autonomy and freedom had been taken away; they believed these measures provoked and legitimized ageism.

Benevolent ageism reflected in the policy recommendations reinforced the stereotype that older adults were not capable of taking care of themselves. Consequently, their autonomy was taken away and they were no longer able to make their own decisions (e.g., to leave the house). One of the Colombian participants used irony to express his frustration about being unnecessary looked after:

Oh no, that you have to take care of them (older adults) (…), at this age one has more responsibility and one knows to care better than any 20‐year‐old kid (…) they annoyed me (…) (CO SH02).

The importance of freedom for participants was discussed by UK M4 as he wanted to have the same rights as the other age population:

I want that freedom. I'd feel that was discrimination against age if we were forced to keep that lockdown longer than other parts of the population.

### Differences between the UK and Colombia

In comparing the two countries, we identified many similarities in the lived experience. However, there were differences in the policies and culture. Whereas the policy to stay indoors in the UK had been strongly advised, the policy in Colombia was implemented in law. The legal enforcement of policy was seen as necessary because cultural respect and trust in the government were absent. CO SHO07 highlighted the wish of older adults to be consulted and respected, remarking:

They (the government) should give us a voice, they have to respect our rights and not steal the money which is meant for the older adults.

In contrast, older adults in the UK were not happy about the advice to stay indoors but followed it to protect themselves and their families.

We suggest that trust in the government is one of the key differences. The participants in both countries demonstrated stoicism, which is represented in the UK in many interviews as “grin and bear it.” The stoicism in the UK remained stable. However, in Colombia, stoicism lessened over time, perhaps reflecting the difference in interview time, as a process of awakening (and mobilization) had begun. Lawyers and influential highly educated people spoke up against the law for older adults to stay at home. This brought much attention towards older adults. The population became aware of stigma against older adults. Participants reflected on their self‐perceptions of ageing and the pandemic. The positive result of legal action made older adults visible. It facilitated older adults to think about themselves as citizens with rights. They believed that their rights violated by limiting their autonomy should be respected. This visibility led to older adults being viewed as a political priority in pandemic policies. CO AH03 reported that:

The pandemic made us visible … The president by a mandatory lockdown also made an acknowledgement that we existed, and later we had to reveal (legal action) ourselves so that they let us release from the lockdown, because it was too excessive (lockdown).

In the UK, by comparison, the homogenous view of older adults persisted. Many participants mentioned that they were not valued by society, but valued by their families. How this will change over time will be examined in the longitudinal follow‐up.

## DISCUSSION

Given the challenging circumstances both of the pandemic itself and the research process, it is remarkable how similar the findings are. The results from the present qualitative study comparing the experiences of older adults from the UK and Colombia highlight how most older adults experienced a new ageism during the pandemic. The impact of COVID‐19 on older adults’ health led politicians, media, and the general public to stereotype older adults as vulnerable and in need of protection. Some participants in both countries experienced the initial COVID‐19 policies as benevolent. The cross‐cultural study allowed us to explore the development of awareness of older adults in Colombia. For the first time, the participants in Colombia felt recognized and, therefore, valued. However, over time many older adults felt exposed to hostile ageism and a loss of autonomy. Many of the UK participants expressed, more often, from the beginning, frustration and anger about the homogeneous view of older adults and their loss of autonomy. Some participants experienced a move from benevolent to hostile ageism over the course of a year. We suggest that a new structural ageism, enabled through COVID‐19, is inherent in both societies and brought to the fore stereotypes of vulnerability and need of protection for older adults. Culturally older adults were seen as homogeneous and with an (enforced) uniform identity.

The findings of decades of gerontological research arguing for the heterogeneity of older adults (Lindenberger et al., 2010) were undermined by society's response to the COVID‐19 pandemic. Ayalon et al. (2020) highlighted the parallel between the COVID‐19 pandemic and an outbreak of ageism. Older adults, aged 70 and over are described in the media as “being helpless, frail, and unable to contribute to society” (p. 1). Thus, the media played a crucial role when communicating the risks of the COVID‐19 pandemic and reporting mostly bad news and the increasing numbers of deaths. These reports propagated the stereotypes of vulnerability, impacting on older adults’ wellbeing and increasing feelings of anxiety and depression. Communication of risk was important but failed when participants stopped watching the news and when they lost trust in the recommended health and safety measures. Thus, media reporting impacts on older adult's well‐being and social identity (Jetten et al., 2020).

**FIGURE 2:**
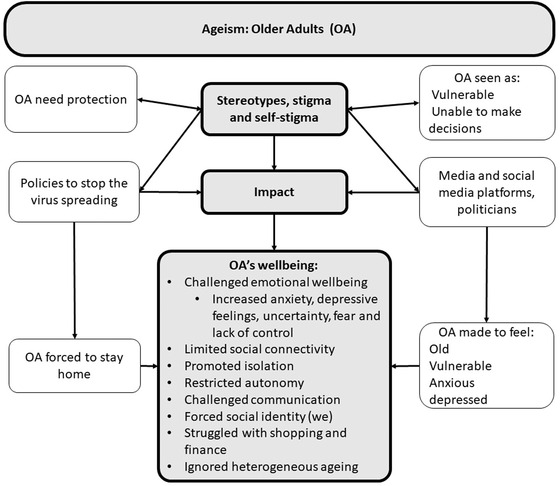
Ageist policies and its impact on older adult's wellbeing

Figure 2 illustrates how the policies of the pandemic impacted on older adult's well‐being. Existing stereotypes, stigma, and self‐stigma about the vulnerability of older adults and their need for protection, both in society and in older people's self‐perceptions, provided the ground for ageist policies. Our findings support the historic analyses of Binstock (2010); when stereotypes influenced policy making in the USA, the existing stereotype of the vulnerability of older adults increased during the COVID‐19 pandemic. However, these new, intended protective, policies challenged older adult's emotional wellbeing, limited social connectivity, and fostered isolation. Although not evident in our data, there is the risk that benevolent ageism might foster cognitive decline (Cohn‐Schwartz et al., 2022; Sublett & Bisconti, 2020; see also Giebel et al., 2021, with respect to dementia). Additionally, the policies restricted individual autonomy, challenged established ways of communication, and consequently, increased anxiety. The increased uncertainty led to fear and lack of control. The forced identity and ignored heterogeneous ageing made many people angry and frustrated (Jetten et al., 2020). Many participants reported support from their family and the positive intergenerational relationships, which helped them to adapt to the restrictions. This finding supports the study from Visintin (2021) who suggested that “positive intergenerational relations are likely beneficial for public health” (p. 1). Many participants talked about COVID‐19 anxiety‐driven behavior (e.g., wearing masks and gloves) to protect themselves. This finding supports Vale et al. (2020) who found that pandemic‐related benevolent ageism led to increasing fear and this fear in consequence motivated people to positive behavioral changes. Swift and Chasteen (2021) claim that the experienced benevolent ageism fosters in society feelings of pity and paternalism, which our participants experienced as a loss of autonomy.

Our study highlights how a new structural discrimination, facilitated by the new COVID‐19 health and safety measures, is present in both countries, reflected in policies, media, and society. Thus, policymakers should be aware of the wider impact of their decisions on people's lives and, in particular, consider whether the changes forced on living circumstances during this and future pandemics might promote ageism. The results of this study should help policymakers develop guidance that respects human rights regardless of age.

## LIMITATIONS AND FUTURE RESEARCH

This study has some limitations. The initial recruitment in the UK was undertaken via Qualtrics, an online platform, which contacted people who had already been recruited to online panels (see McBride et al., 2021 for a fuller discussion). Thus, the UK sample does not include digitally excluded participants. The basic requirements for taking part were phone or online capabilities to engage in the interview itself, although we were able to provide participants with an MS‐Word version of the interview schedule as necessary, and one participant engaged with the study this way. This digital method of recruitment has some advantages. Our UK sample was more stratified compared to other qualitative studies. We were able to recruit the participants more quickly and recruit more men living alone than usual. The participants in Colombia, by contrast, were a convenience sample, so may not be representative of the population, although diversity rather than representativeness is important in qualitative research.

Data collection at different times in the two countries is another limitation of the findings, although this is a problem faced by all cross‐cultural COVID studies. There were potentially additional challenges of conducting interviews remotely. However, in practice, this proved not to be problematic.

Future research is needed to understand how people experienced the consequences of the COVID‐19 pandemic over time between the UK and Colombia. It is important to understand the implications of the lockdown, experienced ageism, and the health and safety measures imposed on older adults. Additionally, research could focus on coping, resilience, and strategies during the post‐pandemic return to normal life. Moreover, it is necessary for future research to include a wider diversity of participants (i.e., gender, class, ethnicity) and adopt an intersectionality approach, as ageing is diverse and multiethnic, and older individuals may experience multiple discriminations.

## CONCLUSION

The COVID‐19 pandemic established a new ageism enabled through the health and safety measures introduced to protect older adults in both the UK and Colombia. We found despite cultural differences between the two countries, the lived experience is similar. Perceived benevolent ageism changed to hostile ageism in Colombia, but in the UK, throughout the pandemic participants experienced mostly hostile ageism. Policy and media established a homogenous view on ageing and ignored heterogenous ageing, which promoted stereotypes. The media continued to report that older adults were at higher risk of getting severely ill, increasing anxiety and frustration among most participants. The loss of autonomy created a move towards respecting the older adults in Colombia, whereas stoicism remained in the UK. The COVID‐19 social policies considered older adults as beneficiaries that means they were receiver and had not been acknowledged as socio‐political individuals who are able to make their own decisions (Arrubla, 2014). For the future, it is important to acknowledge older adults’ responsibility about decision‐making. This decision‐making ability had been taken away from older adults in both countries with the shielding advice in the UK and the law to stay at home in Colombia.

Excluding older adults from participating and decision‐making of policies fosters stereotypes of incapable older adults and proposes old age as an economic burden. This legitimizes ageism and policymakers should be challenged to guarantee inclusive policies to provide well‐being for all ages.

## CONFLICT OF INTEREST

The authors declare that there are no conflicts of interest within this work.

## Notes

1 The term “older adults” in this article refers to all named participants and wider audience of adults ages 60 and over. This reflects the World Health Organization's [WHO] (2021) definition; We are stressing that this term implies the heterogeneity of an ageing population and considers ageing as progress.

2 We use the terms “people” or “person” at some points in the paper, the reason behind is that the public, policy makers, and media used these terms widely and we refer to it during the analyses.
